# Comparative seed germination and seedling development of the ghost orchid, *Dendrophylax lindenii* (Orchidaceae), and molecular identification of its mycorrhizal fungus from South Florida

**DOI:** 10.1093/aob/mcw220

**Published:** 2016-12-26

**Authors:** Nguyen H. Hoang, Michael E. Kane, Ellen N. Radcliffe, Lawrence W. Zettler, Larry W. Richardson

**Affiliations:** 1 Environmental Horticulture Department, University of Florida, PO Box 110675, Gainesville, FL 32611, USA; 2 Department of Plant Biotechnology, University of Sciences, 227 Nguyen Van Cu, Ho Chi Minh City, Vietnam; 3 Orchid Recovery Program, Biology Department, Illinois College, 1101 West College Avenue, Jacksonville, IL 62650, USA and; 4 Florida Panther National Wildlife Refuge, U.S. Fish and Wildlife Service, 12085 SR 29 South Immokalee, FL 34142, USA

**Keywords:** Conservation, flowering, orchid, restoration, reintroduction, mycobiont, *Polyrrhiza lindenii*, leafless

## Abstract

**Background and Aims** The endangered leafless ghost orchid, *Dendrophylax lindenii*, one of the most renowned orchids in the world, is difficult to grow under artificial conditions. Published information on asymbiotic and symbiotic (co-culture with a mycobiont) seed germination, seedling anatomy and developmental morphology of this leafless orchid is completely lacking. This information is critical for the development of efficient procedures for ghost orchid production for successful reintroduction.

**Methods** Ghost orchid seedling early development stages were morphologically and anatomically defined to compare germination, embryo and protocorm maturation and seedling development during asymbiotic and symbiotic culture with one of two mycorrhizal strains (Dlin-379 and Dlin-394) isolated from ghost orchid roots *in situ*.

**Key Results** Seeds symbiotically germinated at higher rates when cultured with fungal strain Dlin-394 than with strain Dlin-379 or asymbiotically on P723 medium during a 10-week culture period. Fungal pelotons were observed in protocorm cells co-cultured with strain Dlin-394 but not Dlin-379. Some 2-year-old seedlings produced multinode inflorescences *in vitro*. Production of keikis from inflorescence nodes indicated the capacity for clonal production in the ghost orchid.

**Conclusions** Ghost orchid embryo and seedling development were characterized into seven stages. Fungal strain Dlin-394 was confirmed as a possible ghost orchid germination mycobiont, which significantly promoted seed germination and seedling development. Internal transcribed spacer sequencing data confirmed that Dlin-394 belongs within the genus *Ceratobasidium*. These results offer the opportunity to examine the benefits of using a mycobiont to enhance *in vitro* germination and possibly *ex vitro* acclimatization and sustainability following outplanting.

## INTRODUCTION

**Fig. 1:**
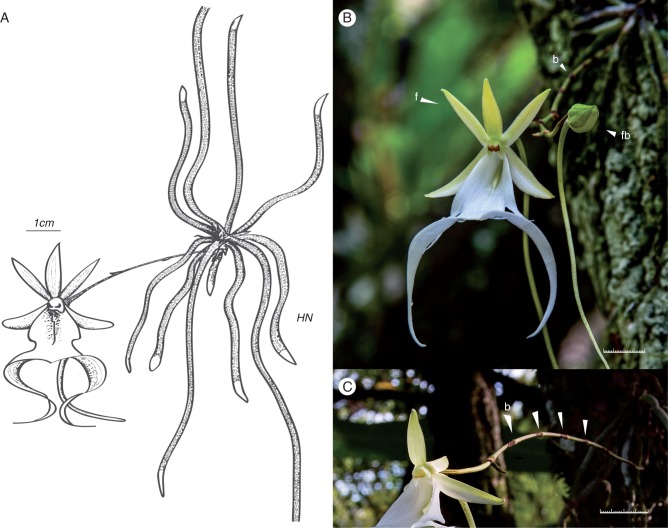
Florida ghost orchid (*Dendrophylax lindenii*) plant and flower. (A) Plant, flower, and two pollinia. (B) Inflorescence with nodal bracts (b), flower (f) and flower bud (fb). (C) Inflorescence with multiple nodal bracts. Scale bar = 1 cm.

The ghost orchid, *Dendrophylax lindenii* (Orchidaceae: Angraecinae), is an endangered New World orchid (Coile and Garland, 1996). This leafless epiphytic species is restricted to small populations in southernmost Florida, Cuba and the Bahamas (Brown, 2002; Rodríguez and Strong, 2012). The ghost orchid consists of a stem with radiating roots appressed to the bark of mostly pond apple (*Annona glabra*), pop ash (*Fraxinus caroliniana*) or swamp cypress (*Taxodium distichum*). The ghost orchid stem is inconspicuous and usually covered with roots or organic debris. Flowering occurs in May–August (Brown, 2002). Flowers are white, showy (Fig. 1A, B) and fragrant at night (Sadler *et al.*, 2011). While no experiment-based information is available, ghost orchid plants are reported to be very challenging to grow under greenhouse conditions (Davis, 2009*a*, *b*; Mirenda, 2013). With their high public profile (Kaufman *et al.*, 2003; Orlean, 2011), ghost orchids often become the target of poaching (Brown, 2002), even in areas protected by law in both Florida and Cuba (E. Mujica, Orquideario Soroa, Pinar del Rio, Cuba and M. Owen, Fakahatchee Strand Preserve State Park, USA, pers. comm.). *Dendrophylax lindenii* populations are also threatened by phytophagous pests (Zettler *et al.*, 2012) and wetland hydrological changes (Langdon, 1979; Coile and Garland, 2003). Moreover, most *D. lindenii* populations lie within low-lying coastal areas that are vulnerable to periodic hurricanes (Wiegand *et al.*, 2013). Raventós *et al.* (2015) reported that *D. lindenii* in Cuba could become extinct within 25 years if the annual probability of disturbances, including hurricanes, exceeds 14 %. Despite the threatened status of the species and many efforts to grow it, there is currently no scientific literature on ghost orchid conservation, *in vitro* germination or the importance of mycorrhizal fungi.

Like the seeds of other orchid species, those of the ghost orchid are presumably dependent upon mycorrhizal fungus infection for germination in nature (Rasmussen *et al.*, 2015). Orchid seeds contain rudimentary embryos bearing little reserves, mostly lipid or protein (Arditti, 1992). However, sugar and starch deposits have been documented in a few orchid species (Manning and Van, 1987; Yeung and Law, 1992). Fungal mycobionts supply water and nutrients, such as minerals, sugar, thiamine and folic acid, which promote germination and protocorm development (Arditti, 1967; Hijner and Arditti, 1973; Rasmussen, 1995; Bougoure *et al.*, 2014). Numerous symbiotic and asymbiotic studies have been conducted to investigate orchid seeds’ germination requirements (reviewed by Arditti, 2009). Quantification of seedling development in many leafy orchids has made it possible to precisely evaluate seed germination methods and to characterize seedling developmental stages (e.g. Kauth *et al.*, 2011; Teixeira da Silva *et al.*, 2015). To evaluate different germination procedures for leafless orchids, a highly modified orchid group (Carlsward, 2004), their seedling developmental stages under *in vitro* conditions need to be described in detail.

Little is known about orchid seed germination niche requirements *in situ*, especially the role and host specificity of mycorrhizal fungi during germination and through subsequent seedling development (Stewart and Kane, 2007; Rasmussen *et al.*, 2015). *Rhizoctonia*-like fungi *Ceratobasidium*, *Sebacina* and *Tulasnella* are three major genera that form mycorrhizae with orchid species (Yukawa *et al.*, 2009). *Ceratobasidium* fungi form mycorrhizae with the leafless orchid genus *Campylocentrum* (Richardson *et al.*, 1993; Otero *et al.*, 2002), a genus closely related to *Dendrophylax* (Carlsward *et al.*, 2006*b*). Chomicki *et al.* (2014) suggested that the fungal pelotons in *D. lindenii* roots morphologically resemble *Ceratobasidium* pelotons. However, Yokoya *et al.* (2015) isolated *Tulasnella* fungal strains from the Angraecinae species *Angraecum magdalenae* and *Angraecum* *protensum.* The confirmation of Chomicki’s hypothesis therefore requires fungal isolation, molecular identification and examination of symbiotic germination.

Currently, we are not aware of any published reports of the *in vitro* seed germination of the ghost orchid alone and with its mycobionts. Such information is required to guide sound management strategies for successful reintroduction of *D. lindenii*. To our knowledge, this is the first report describing *D. lindenii* seedling developmental stages, fungal isolation, molecular identification and mycorrhizal verification of *Rhizoctonia*-like fungi by means of symbiotic seed germination.

## MATERIALS AND METHODS

### Study site

Seeds and roots of *Dendrophylax lindenii* were acquired from the Florida Panther National Wildlife Refuge (FPNWR), a 10 684-ha area located in remote north central Collier County, FL, known to harbour 27 orchid species in 17 genera (Stewart and Richardson, 2008). Typical of the Big Cypress Region ecosystem, most of the epiphytic taxa found within the FPNWR are confined to patches or ‘islands’ of strand swamps and sloughs shaded by an upper canopy of bald cypress (*Taxodium distichum*). Beneath this canopy, the majority of epiphytic orchids are affixed to branches of mature pop ash (*Fraxinus caroliniana*) and pond apple (*Annona glabra*) that overhang pools of stagnant water. Epiphytic orchid associates of *D. lindenii* include *Epidendrum amphistomum* (dingy-flowered star orchid), *Epidendrum* *nocturnum* (night-scented orchid), *Epidendrum* *rigidum* (rigid epidendrum), *Polystachya concreta* (yellow helmet orchid) and *Prosthechea cochleata* var. *triandra* (Florida clamshell orchid). Two other leafless epiphytes are also present: *Campylocentrum pachyrrhizum* (ribbon orchid) and *Dendrophylax porrectus* (jingle bell orchid). All of these species, including *D. lindenii*, are listed as ‘endangered’ on Florida’s Regulated Plant Index (Coile and Garland, 1996).

### Orchid seed source

Seed capsules were collected at Cochran Lake (FPNWR, Naples, FL, USA), an isolated strand swamp island harbouring a population of ∼80 *D.* *lindenii* individuals, of which 5–10 % flower each summer. Ghost orchid capsule production in nature is very rare. Seeds were obtained from two mature capsules harvested in March 2013 and June 2014. Seeds from the March 2013 capsule were used to characterize seedling developmental stages. Capsules were the result of artificial hand pollinations, with pollen donated from separate nearby individuals. Capsules were promptly (<24 h) dried at ambient temperature (22 ± 2 °C) over CaSO_4_ desiccant (Drierite, W.A. Hammond Co., Xenia, Ohio, USA) for 15 d until thoroughly dry, then stored in darkness at −10 °C until use.

Root samples that yielded mycorrhizal fungi were obtained from West Hinson Lake, a separate strand swamp island located ∼1 km south-west of Cochran Lake. The first and second samples were collected on 19 June 2013 and 31 July 2014, respectively, from two separate individuals. Collection consisted of detachment of the tip (1–4 cm segment) of an actively growing root firmly affixed to the host tree substrate on one side. Root samples were gently removed from the bark substrate by means of a sterile scalpel. Each root segment was placed into a sterile plastic bag and refrigerated (∼4 °C) within 2 h of collection until fungal isolations were performed (24–48 h after collection).

### Fungal isolation, preliminary identification and storage

Mycorrhizal fungi were isolated from the root cortical regions using standard protocols applied to other epiphytic orchids, such as those described by Richardson *et al.* (1993), Zettler *et al.* (2013) and Yokoya *et al.* (2015). Briefly, roots were removed from refrigeration and rinsed with sterile deionized water, and the epidermis was gently scraped to remove surface debris. Roots were then measured and photographed to facilitate eventual documentation and location of pelotons along their length. Roots were surface-sterilized for 1 min in a solution consisting of 90 mL of sterile water, 5 mL of Clorox^®^ (8·25 % NaOCl) and 5 mL of 100 % ethanol (95 %), followed by two 1-min rinses in sterile deionized water. Beginning at the tip, roots were cut into pieces 1 cm long, and each piece was placed into a separate sterile 9-cm diameter Petri dish containing a 5-mL drop of sterile deionized water. Clumps of cortical cells harbouring fungal pelotons were macerated and teased apart using a sterile scalpel and forceps within the 5-mL deionized water droplet. Molten (warm) Fungal Isolation Medium (FIM) containing streptomycin sulphate antibiotic (Clements and Ellyard, 1979) was slowly added to the droplet, and the agar/cortical cell mixture was gently swirled in a circular motion to facilitate separation of cortical cells, then allowed to cool and solidify. Using a dissection microscope, plates were inspected 24–48 h later for signs of active hyphal growth emerging from pelotons. To quantify peloton number per plate (segment), a quadrant was drawn with a thin-tip black Sharpie permanent pen (Shelbyville, TN, USA) on the bottom of the plate, sectioning the plate into four quadrants. Pelotons were then circled on each plate using the Sharpie, and totals were recorded. Pelotons and/or hyphal tips emanating from pelotons were subcultured onto potato dextrose agar (PDA, Difco, Becton Dickinson and Co., Sparks, MD, USA) using a sterile scalpel. The plates were incubated at ambient temperature.

Orchid mycorrhizal fungi were distinguished from common moulds using previously published descriptions (Currah *et al.*, 1987, 1990; Richardson *et al.*, 1993). Fungi that yielded morphological characteristics (e.g. monilioid cells) resembling basidiomycetes in the *Rhizoctonia* complex (e.g. Tulasnellaceae, Ceratobasidiaceae) were retained for further identification using molecular techniques. Subcultures of important strains were transferred to new PDA plates every 2 months and maintained at ambient temperature, and backup cultures were retained on an oat-based medium [2·5 g rolled oats, 7·0 g agar, 1 L deionized water (Dixon, 1987) at 4 °C refrigeration]. To safeguard these strains for future work and conservation, some subcultures were also deposited in the University of Alberta Microfungus Collection and Herbarium (UAMH), Edmonton, Canada.

### Molecular identification of fungi

Molecular identification followed the procedures outlined in Zettler *et al.* (2013) and Yokoya *et al.* (2015), involving ribosomal DNA internal transcribed spacer (ITS) amplification and Sanger sequencing. This consisted of initially growing a strain in liquid media (FIM broth without agar or streptomycin) on a shaker at ambient temperature until harvesting, ∼1–2 months after inoculation. An Omega E.Z.N.A.^®^ Fungal DNA Mini Kit (Omega Bio-Tek Inc., Norcross, GA, USA) was used to isolate genomic DNA from mycelia. The ITS regions were amplified using primers ITS1-OF-T and ITS4-OF and an Omega E.Z.N.A.^®^ Fungal DNA Mini Kit (Omega Bio-Tek Inc., Norcross, GA, USA). The reactions were performed in a programmable thermocycler for an initial denaturation at 94 °C for 5 min, 45 cycles of denaturation at 94 °C for 30 s, annealing at 38  C for 30 s and extension at 72 °C for 1 min, and a final extension step at 72 °C for 5 min. Amplification products were verified by electrophoresis on 2 % agarose gels containing 0·1 mg mL^−1^ ethidium bromide. All sequence analyses were sent to the University of Illinois UIUC Core Sequencing Facility. The forward and reverse sequences and chromatograms were checked for accuracy and consensus and were compared with database sequences using BLAST (National Center for Biotechnology Information, Bethesda, MD, USA).

### Asymbiotic seed germination for seedling developmental stage description

In February 2014, ghost orchid seeds were surface-sterilized in a solution containing 90 mL of sterile water, 5 mL of Clorox^®^ (8·25 % NaOCl) and 5 mL of 100 % ethanol for 1 min, and then rinsed twice with sterile distilled deionized water with seed being concentrated in a centrifuge at 1914 *g* (4000 rpm with 10.7 cm radius rotor). Sterilized seeds were germinated in 100 ×15-mm Petri plates (∼200 seeds/plate) containing 25 mL of P723 orchid seed sowing medium (P723, PhytoTechnology Laboratories^®^, Shawnee Mission, KS, USA), a modified quarter-strength MS-based medium that is broadly used for orchid seed sowing. The inoculated plates were transferred to a Percival 136LL incubator (Percival Scientific, Inc., Perry, IA, USA) maintained at 25 °C under a 16-h light/8-h dark photoperiod provided by cool white fluorescence tubes (GE F20T12-CW) at 36 µmol m^−^^2^ s^−^^1^. Seedlings were collected 0, 3, 5, 7, 10, 15, 20, 25, 30, 35, 40, 45, 50, 55, 60 and 80 d after germination (DAG) for observation and histological fixing.

#### Morphological observations

 The morphological features of ungerminated seeds and developing seedlings over time were documented for a period of 80 DAG. In addition, morphological and anatomical characteristics of 2-year-old seedlings, maintained *in vitro*, were also examined. Specimens were photographed using a Nikon Alphaphot YS2 microscope and Carl Zeiss Tessovar stereoscope. Seed and seedlings were fixed and stored in formalin–acetic–alcohol (FAA) and then dehydrated in a 70 %, 95 %, 100 % graded alcohol series. Dehydrated samples were critical point dried (Denton Vacuum LLC NJ Desk V) and then sputter-coated with gold/palladium before being mounted and observed in a scanning electron microscope (SEM) system (Hitachi High Technologies America S4000 Series FE) at the Interdisciplinary Center for Biotechnology Research, Electron Microscopy Core (University of Florida).

#### Histological sectioning

 Anatomical studies were conducted using seedling tissues preserved in FAA. Samples were dehydrated using an ethanol and *t*-butanol mixtures series before embedding in paraffin (T565, TissuePrep^®^). Tissue sections of 5 µm thickness were cut on a Leica rotary microtome, stained with safranin/fast green and then mounted using Permount^®^.

### Comparative asymbiotic and symbiotic seed germination

Orchid seeds were surface-sterilized using the procedure described above. Sterilized seed density was diluted to an average of 45 seeds per 50 µL. Actual seed numbers ranged from 19 (minimum) to 119 (maximum). A 50-µL seed suspension aliquot was dispensed onto each Petri plate containing 25 mL of P723 medium, oatmeal agar medium (OM) without fungus (asymbiotic control) or OM with Dlin-394 or Dlin-379. Sterile 1×4-cm Fisherbrand™ P8 filter paper strips (09-795C, Fisher Scientific, Pittsburgh, PA, USA) served as the support for inoculated seed in Petri plates containing OM. A 1×1-cm agar block cut from a 10-d-old fungal culture was transferred to the centre of the Petri plate and served as the fungal source for symbiotic germination. Petri plates containing P723 or OM medium but no fungus served as control treatments. After inoculation, plates were sealed with a layer of PVC sealing film (A003, PhytoTechnology Laboratories^®^, Shawnee Mission, KS, USA) and transferred to an incubator maintained at 25 °C under a 16-h light/8-h dark photoperiod provided by cool white fluorescence tubes (GE F20T12-CW) at 40 µmol m^−^^2^ s^−^^1^. The position of each replicate Petri plate of the four treatments was randomized within the incubator. Seed germination percentages and the frequency of seedlings at each development stage were recorded weekly for 10 weeks. Final germination was calculated as the total percentage of seedlings in stages 3–6. The experiment was repeated once.

### Statistical analysis

Differences in germination rate between germination methods were determined using logistic regression, the GLIMMIX procedure (SAS^®^ 9.2) and Tukey’s *post hoc* multiple comparison adjustment (α = 0·05) was used for all pairwise comparisons of method means.

## RESULTS

### Fungal isolation and identification

**Fig. 2:**
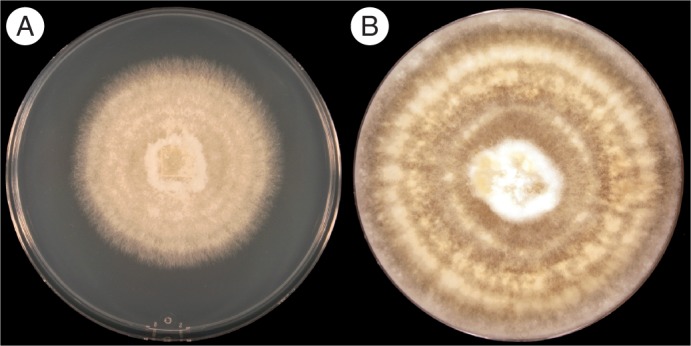
Initial and aged cultures of *Ceratobasidium* strain Dlin-394 on potato dextrose agar (PDA) plate (diameter 10 cm). (A) Initial (7 d). (B) Aged (14 d). Concentric rings are clearly visible in the centre of the plate. Note the raised fluffy aerial mycelium along the sides of the plate, typical of many *Ceratobasidium* cultures.

Pelotons of *D. lindenii* were detected in actively growing roots of mature plants 2 cm from the tip of the root. Root tips (1 cm) and regions beyond the second centimetre point did not harbour detectable pelotons in the samples. On PDA, all pelotons yielded *Rhizoctonia*-like fungi that fitted the typical profile of orchid mycorrhizal fungi in culture, namely strains of *Ceratobasidium* (anamorphs formally classified as *Ceratorhiza* Moore). These cultures exhibited yellowish to tan-coloured colonies with rapid growth rates (0·15–0·25 mm h^−1^ on PDA at 22 °C). Initial growth consisted of submerged/surface mycelium with noticeable concentric rings (zonation is shown in Fig. 2A), followed by fluffy aerial mycelial tufts on aged (>14 d) colonies that grew up and over the outer edge of the Petri dish (Fig. 2B). Under light microscopy, barrel-shaped to elliptical monilioid cells were evident. Two strains of *Ceratobasidium* were isolated in both years (Dlin-379, Dlin-394), and one (Dlin-379) has since been deposited in the University of Alberta Microfungus Collection and Herbarium (Canada) as UAMH 11750. The second strain (Dlin-394) has yet to be deposited in UAMH and assigned an accession number; subcultures of both strains are also maintained in storage (4 °C) at both institutions (Illinois College and University of Florida). Sequencing of the ITS region of ribosomal DNA from multiple cultures of the isolate confirmed that the *D. lindenii* *strain* Dlin-394 was indeed *Ceratobasidium*. There were 571 identical bases over a total of 616 bases, giving 93 % similarity. However, ITS sequencing did not allow identification to the species level. Results obtained did not match the sequences of any accession in the NCBI database, despite repetition of the DNA extraction and sequencing of this particular strain.

### Ghost orchid germination and seedling development

**Fig. 3:**
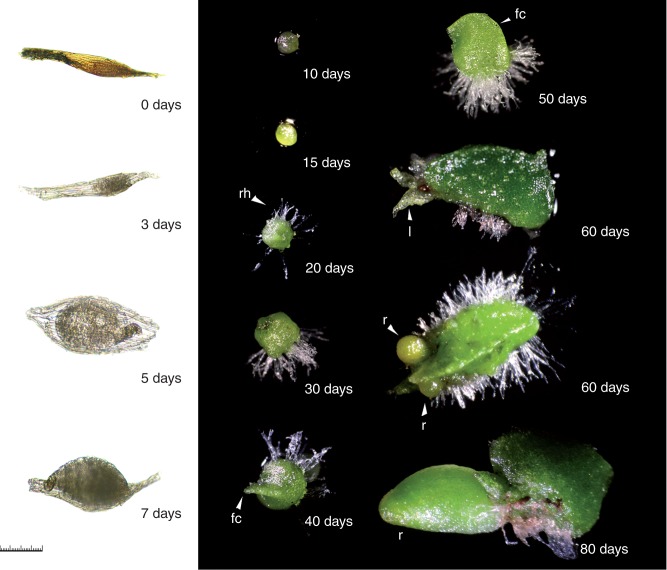
Ghost orchid asymbiotic embryo and seedling development from 0 to 80 d after germination (DAG). (0 DAG) ungerminated seed; (3–5 DAG) seed with swollen embryo; (7 DAG) differentiating embryo with ruptured testa; (10–15 DAG) embryo enlargement; (20–30 DAG) rhizoid formation; (40 DAG) formation of the dorsal crest (dc); (50 DAG) elongation of the dorsal crest; (60 DAG) Emerging leaves (l) and roots (r); (80 DAG) seedling with elongated root. Scale bar = 100 µm (0–7 DAG) or 500 µm (10–80 DAG).

**Fig. 4:**
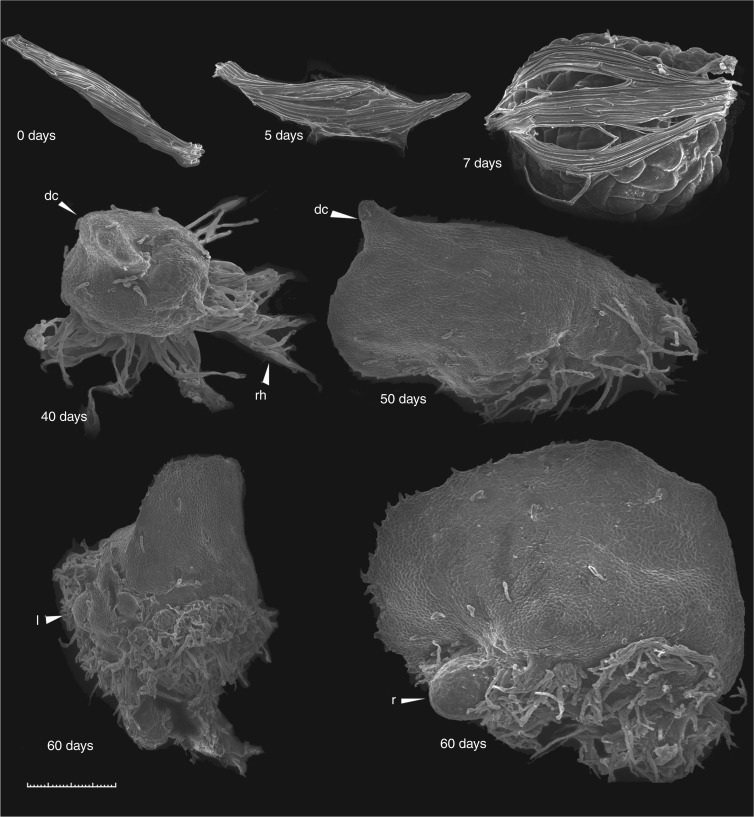
Ghost orchid embryo and seedling development from 0 to 60 d after germination (DAG) under SEM. (0 DAG) ungerminated seed; (5 DAG) seed with swollen embryo and partially ruptured testa; (7 DAG) enlarging embryo with ruptured testa; (40 DAG) differentiating embryo with dorsal crest (dc) and rhizoids (rh); (50 DAG) differentiating embryo with elongated dorsal crest; (60 DAG) seedling with shoot and emerging leaves (l) or roots (r). Seedlings with elongated roots at 80 DAG were not observed under SEM due to specimen size limitations. Scale bar = 100 µm (0–7 DAG) or 500 µm (40–60 DAG).

**Fig. 5:**
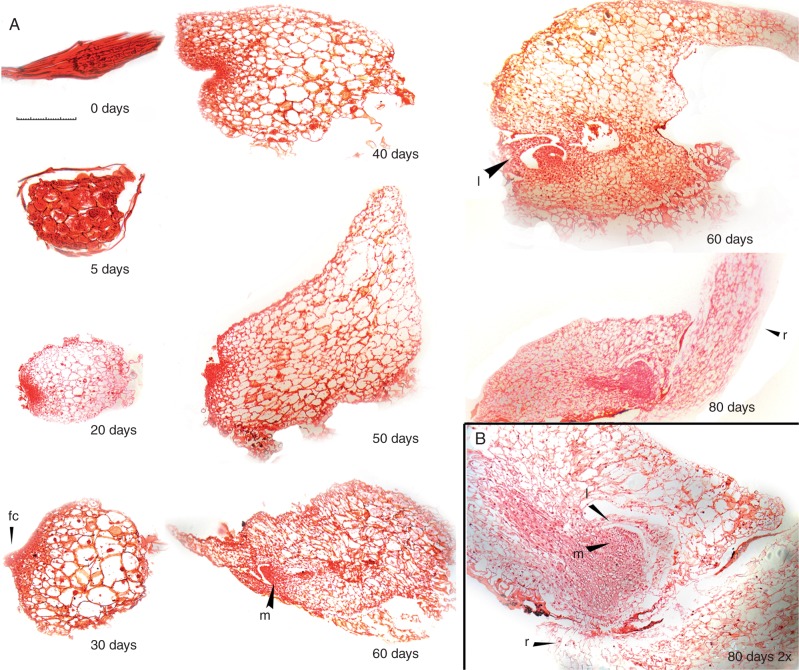
Histological analysis of safranin/fast green-stained embryo and seedling sections from 0 to 80 DAG. (A) 0–80 DAG. (0 DAG) ungerminated seed; (5 DAG) seed with swollen underdeveloped embryo; (20 DAG) enlarging embryo with ruptured testa and meristematic tissue formation; (30 DAG) differentiating embryo with the dorsal crest (dc) emerging; (40–50 DAG) protocorm with elongating dorsal crest; (60 DAG) Differentiating embryo with meristem (m) and leaves (l); (80 DAG) seedling with elongated root. (B) Magnified meristem with root formation at the base. Scale bar = 100 µm (0–5 DAG), 500 µm (20–80 DAG) or 250 µm (80 DAG, magnification 2× scale bar).

Seed germination, sequential changes in seedling developmental morphology, and anatomy were described. Dehydrated seed stored at −10 °C measured 327 ×46 µm (average of ten seeds) and were brown in colour (0 DAG; Figs 3–5). The testa surrounding ungerminated seeds was highly elongated (three to five cells along the longitudinal axis), forming a distinct marginal ridge (0 DAG; Figs 3 and 4). During surface sterilization in dilute sodium hypochlorite, seeds became bleached, losing their brown colour. Seed imbibition was observed after 3 d of culture (3 DAG; Fig. 3). By 5 DAG embryos had become swollen (Fig. 3–5) and minor testa rupture was noted (5 DAG; Fig. 4). By day 7 embryos had enlarged rapidly, resulting in major testa rupture (7 DAG; Figs 3–5). Embryos initially developed into globular protocorms and continued to increase in diameter, forming light green globular protocorms (7–30 DAG; Fig. 3). Rhizoids first developed along the protocorm base by 20 DAG (Fig. 3).

Embryo polarity was established early, as noted by development of the protomeristem at the apex by 20 DAG (Fig. 5). During 30–50 DAG, a dorsal crest (Veyret, 1974), also described as the first leaf (Nishimura, 1981), developed from this meristematic region and continued to elongate (Figs 3–5), resulting in an embryo with distinct dorsiventral symmetry. By 60 DAG a small number of protocorms with shoot meristems bearing reduced leaves were observed (Figs 3 and 4). However, on most protocorms surface protrusion of shoots and leaves was not observed. Histological sectioning revealed that fully developed shoot meristems bearing leaves were either completely enclosed within the protocorm or partially emergent (60 DAG; Fig. 5). Concurrently, the first visible root was observed to emerge from the protocorm base (60 DAG; Figs 3–5). Roots subsequently originated endogenously from the bases of internal shoot meristem bearing leaves. Roots elongated and became highly chlorophyllous by 80 DAG (Figs 3 and 5). A vascular connection between the shoot meristem and the protocorm was observed by 80 DAG (Fig. 5A, B). Based upon our morphological and histological analysis, seven seedling developmental stages were defined (Table 1). These described stages were used to numerically compare effects of asymbiotic and symbiotic seed culture on ghost orchid seed germination and seedling development.

**Table 1.** Ghost orchid seedling development stages

Stage	Description
0	Ungerminated seed with embryo
1	Seed with swollen embryo
2	Enlarged green embryo with ruptured testa
3	Differentiating embryo with trichomes or dorsal crest tip
4	Differentiating embryo with elongated dorsal crest
5	Seedling with emerging leaf primordia or first root
6	Seedling with elongated root(s)

**Fig. 6:**
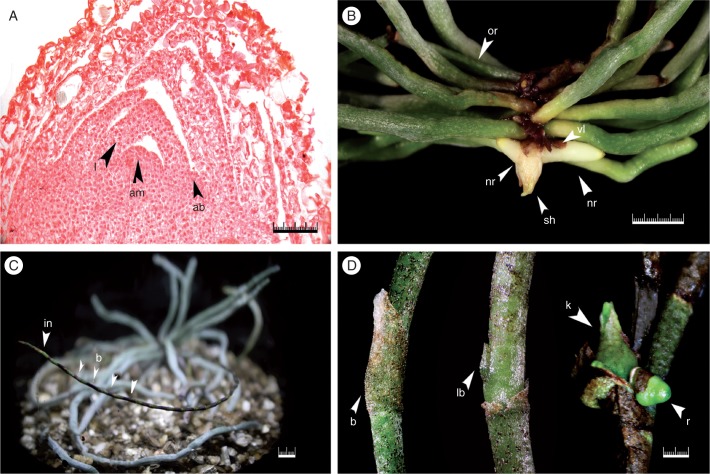
Shoot meristem and floral stalk node. (A) Mature seedling shoot meristem consisting of apical meristem (am), leaves (l) and axillary bud (ab). (B) Two-year-old seedling with vestigial leaves (vl) and new root (nr) formed adjacent to the apical shoot (sh). Old roots (or) are located at the basal end of the reduced shoot apex. (C) Two-year-old seedling with inflorescence (in) produced *in vitro* containing multiple lateral buds covered by nodal bracts (b). (D) Nodal bract (b) covering lateral bud (lb). Keiki (k) and root (r) formation on cultured excised inflorescence nodal explant. Scale bar = 0·5 mm (A), 1 cm (B, C), 1 mm (D).

Two-year-old seedlings maintained *in vitro* consist of a reduced stem with lateral shoot meristems bearing reduced leaves (Fig. 6A) and multiple roots covered by a thick velamen developing behind the root apex. Similar to root formation in protocorms, new roots originated from the tissue subtending the shoot meristems (Fig. 6B). Some mature seedlings developed multi-node inflorescences *in vitro* (Fig. 6B, C). All inflorescences developed quickly, but became brown and died after 2–3 months. A few inflorescences exhibited indeterminate growth consisting of >20 nodes (Fig. 6C). Removal of the nodal scales revealed the presence of underlying lateral buds (Fig. 6D). Hand dissection of these buds or the inflorescence tip did not reveal the presence of floral structures. Formation of keikis (plantlets) from cultured excised inflorescence nodes was observed (Fig. 6D).

### Comparative symbiotic and asymbiotic seed germination

**Fig. 7:**
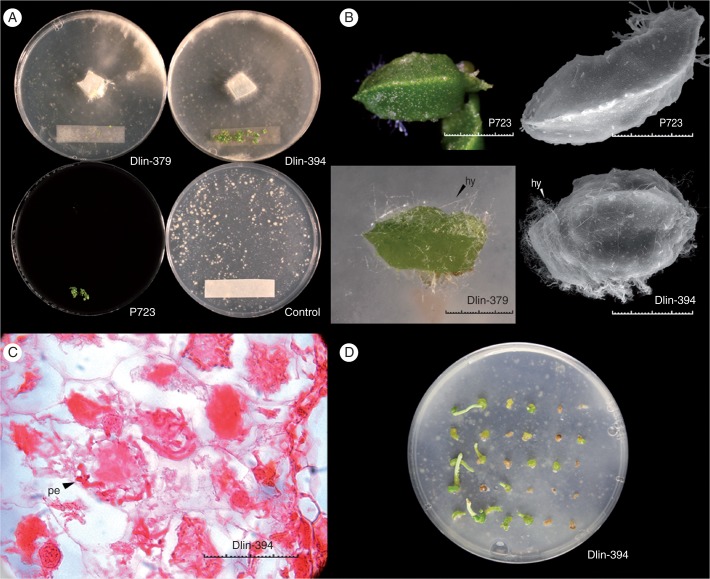
Comparative asymbiotic and symbiotic ghost orchid seed germination. (A) Ten-week-old orchid seeds and seedlings from four treatments. (B) Ten-week-old asymbiotic seedlings and symbiotic seedlings. (Top row) Seedling from P723 medium. (Lower row) Seedling infected with Dlin-394 hyphae (hy). (C) Peloton (pe) formation was observed in seedlings co-cultured with fungal strain Dlin-394. (D) Seedlings co-cultured with Dlin-394 for 10 weeks from germination followed by 10 weeks of subculture on OM. Petri plate diameter = 10 cm (A, D). Scale bar = 1 mm (B), 0·05 mm (C).

The seed germination stages defined from the experiment above (Table 1) were used to evaluate the effectiveness of the different seed germination methods using P723 (asymbiotic), OM alone (OM control) or OM with Dlin-394 or Dlin-379 (symbiotic; Fig. 7A). Within 10 weeks, ghost orchid seedlings developed to stage 5 on P723 medium or on OM when co-cultured with Dlin-394 (Fig. 7B). Histological screening verified the absence of pelotons in asymbiotically germinated protocorms, whereas many pelotons with associated mycelia were observed in symbiotically germinated protocorms co-cultured with the Dlin-394 fungal isolate (Fig. 7C).

**Fig. 8:**
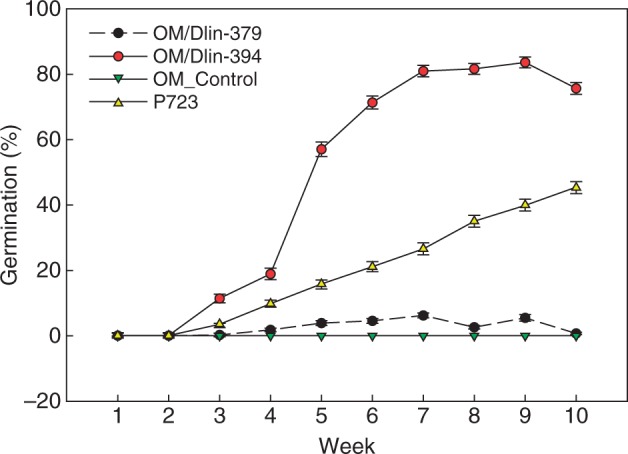
Comparative effects of two fungal strains, Dlin-379 or Dlin-394, on weekly seed germination percentages during symbiotic culture on oatmeal agar medium (OM) or asymbiotic culture on P723 medium for 10 weeks. Results represent the mean responses of two repeated experiments.

After 2 weeks, seeds co-cultured with Dlin-394 germinated at a significantly higher rate than those on P723 medium alone. Dlin-379 co-culture did not promote seed germination under the experimental conditions tested. On OM alone (asymbiotic OM control), seeds imbibed water and expanded with testa rupture, but embryos did not develop further. Seed infected with Dlin-394 germinated to a maximum of 84 ± 2 % by week 9. By week 10, total germination had decreased to 76 % following abrupt browning and death of protocorms. Percentage seedling germination on P723 was lower than with seed co-cultured with Dlin-394, but total germination increased over time (Fig. 8). Assessment of initial germination responses was terminated at 10 weeks, when protocorms were transferred onto fresh medium. Total germination percentage was significantly different between P723 (45 ± 2 %), OM/Dlin-394 (76 ± 2 %), OM/Dlin-379 (0·6 ± 0·3 %) and OM alone (1·0 ± 0·1 %) (Fig. 8).

**Fig. 9:**
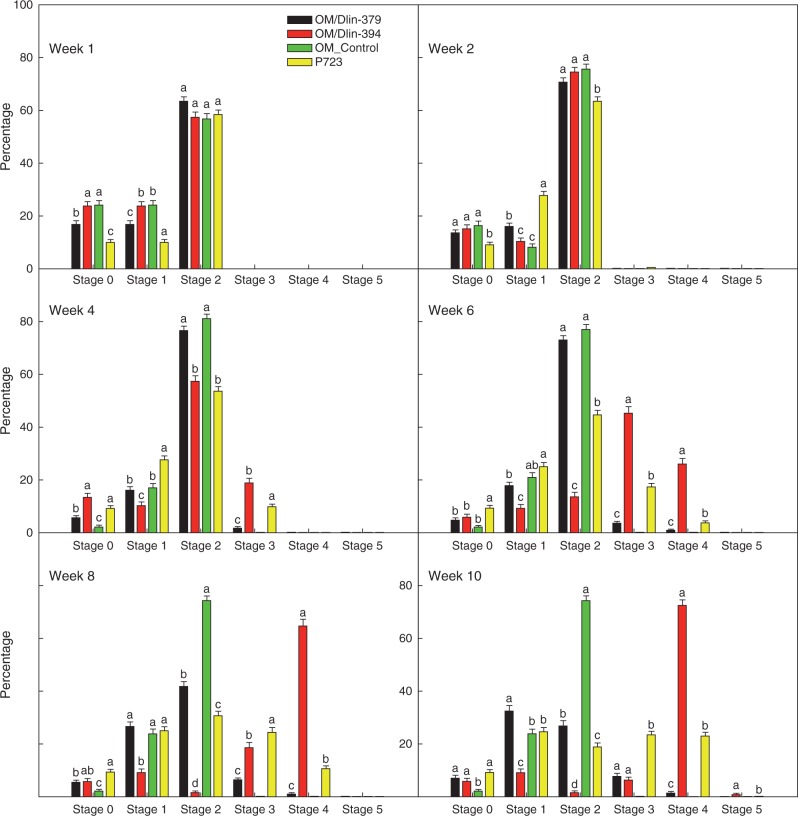
Comparative effects of two fungal strains, Dlin-379 or Dlin-394, on seedling development stage during symbiotic culture on oatmeal agar medium (OM) or asymbiotic culture on P723 medium for 10 weeks. Refer to Table 1 for specific developmental stage characteristics. Results represent the mean responses of two repeated experiments. Treatments with different letters are significantly different within each stage at α = 0·05.

Within the first week of germination, seeds in all four treatments imbibed and developed to stage 2 at similar rates (57–63 %) (Fig. 9; week 1). After 2 weeks, the percentage of stage 2 seedlings continued to increase (63–75 %) except for seedlings asymbiotically cultured on P723 (Fig. 9; week 2). Germinated seeds became chlorophyllous on both P723 and OM/Dlin-394. By week 4, the percentage of stage 3 seedlings with rhizoids or dorsal crest tips was significantly higher on OM-394 (Fig. 9; week 4). The percentages of stage 4 seedlings on OM/Dlin-394 and P723 (asymbiotic culture) increased from week 6 to week 10 and were consistently higher on OM/Dlin-394. In contrast, on OM/Dlin-379 a very low percentage of seedlings developed to stage 4 by week 10 (Fig. 9). After week 7, many protocorms produced on OM/Dlin-379 turned brown and died, resulting in a decrease in the percentage of seedlings attaining stage 2 (Fig. 9; weeks 8–10). Some protocorms infected with Dlin-394 also became brown and died, but only after 9 weeks of culture. Protocorms initially infected with Dlin-394 exhibited variable growth and development patterns, including mortality, after being transferred onto fresh OM for an additional 10 weeks (Fig. 7D). In addition, some seedlings developed to stage 6, bearing elongated roots (Fig. 7D).

## DISCUSSION

### Fungus isolation and identification

The ITS sequencing of Dlin-394, with germination promotion capacity, confirmed the isolate to be a *Ceratobasidium* species (Fig. 2). The ITS sequences for *Ceratobasidium* Dlin-394 did not match any specific accession in the NCBI database, despite repetition of amplification and sequencing. This result suggested that *Ceratobasidium* Dlin-394 may be a unique, heretofore uncharacterized mycorrhizal fungus.

Since protocorms of this threatened species were not available in the field, both fungal strains used in this experiment were isolated from adult ghost orchid roots, which is not uncommon in many orchid mycorrhizae studies (Smith and Read, 1996; Zettler and Hofer, 1998; Zettler *et al.*, 2007). For example, Kristiansen *et al.* (2001) found two different mycorrhizal taxa from the same single peloton in a *Dactylorhiza majalis* root cell. Likewise, Taylor and Bruns (1997) isolated different fungal strains from different *Corallorhiza maculata* growing nearby. Even though both fungi were isolated from mature ghost orchid roots, their roles as seed germination mycobionts are not guaranteed until a symbiotic germination experiment is conducted.

Dlin-394 was isolated from adult ghost orchid roots, confirming Chomicki’s hypothesis that adult ghost orchids do associate with a *Ceratobasidium* fungus (Chomicki *et al.*, 2014). In accordance with this result, *Ceratobasidium* was also isolated from many Vandae epiphytics, such as *Campylocentrum* spp. (Otero *et al.*, 2002; Radcliffe *et al.*, 2015), *Aerangis* spp. and *Taeniophyllum obtusum* (Irawati, 2009). Our verification of *Ceratobasidium* from *D. lindenii* in South Florida lends support for Yukawa’s view (2009) that members of the Ceratobasidiaceae are linked to orchids in the Vandeae tribe, especially the Angraecinae subtribe, by a ‘phylogenetic signal’ (Yukawa *et al.*, 2009; Rasmussen and Rasmussen, 2014). Furthermore, Chomicki *et al.* (2014) found mycorrhizal structures (pelotons) in various states of digestion in adult ghost orchid roots, and a specialized mechanism for restricting fungal growth in the lower cortex without reducing photosynthesis in the upper root layer, thereby maximizing carbon gain. These results, coupled with the seed germination promotion capacity of *Ceratobasidium* Dlin-394, suggested a high level of fungal dependence during both early and adult stages of the ghost orchids *in situ*.

Interestingly, the *Ceratobasidium* strains isolated from co-habiting ribbon orchids (*C. pachyrrhizum*) from the same area (FPNWR) are different from the ghost orchid strain (Dlin-394), based on BLAST results (E. N. Raadcliffe, unpubl. res.). Thus, it is conceivable that the ghost orchid may also display some degree of fungal specificity, justifying further inquiry. Sequencing of the ITS region of future mycorrhizal isolates from *D. lindenii* is planned, and should facilitate comparison with, and identification of, the mycorrhizae between orchid populations within Florida and between Florida and Cuba.

### Seedling development

Orchid embryos display very limited histodifferentiation while enclosed within the testa and display a special type of morphological dormancy (Baskin and Baskin, 2014). Post-germination embryo maturation and seedling development occur outside of the seed coat following embryo imbibition, swelling and rupture of the seed coat (Arditti, 1967). The freed embryo is described as the ‘protocorm’ (Arditti, 1992) and has been described as morphologically similar to *Lycopodium* globular embryos (Arditti, 1992), although their developmental pathways are completely different. Whether protocorms are differentiating germinated embryos or underdeveloped germinated seedlings is a source of contention. Some orchidologists regard protocorms, bearing shoot and root proto-meristems, as underdeveloped seedlings because of their bipolarity (Batygina and Andronova, 2000; Batygina *et al.*, 2003). Nishimura (1981) observed that some Vandeae protocorms resemble modified first leaves, having the capacity for photosynthesis, and similarly concluded that protocorms were seedlings. However, following seed coat separation, orchid embryos are generally considered as developmentally immature (Arditti, 1992). While differentiation between the terms orchid ‘protocorm’, ‘embryo’ and ‘seedling’ in current literature is confusing, we believe that ghost orchid protocorms in the globular stage (stages 1 and 2) are still undergoing embryo development, which culminates first in the production of a *mature embryo*, consisting of a shoot meristem with true leaves and a subtending root meristem. We consider the emergence of the first root to represent the beginning of the true *seedling stage* (stage 5; Figs 3 and 4).

Orchid seedling development *in vitro* has been morphologically categorized into stages in various leafy terrestrial and epiphytic orchids, such as *Calopogon* (Kauth *et al.*, 2011), *Bletia* (Johnson *et al.*, 2011), *Habenaria* (Stewart and Kane, 2006), *Spiranthes* (Zettler *et al.*, 1995) and *Cyrtopodium* (Dutra *et al.*, 2009). Categorization of development stages allows seedling development to be measured and the efficacy of germination procedures to be compared. However, unlike most leafy orchids, leafless orchids, like *D.* *lindenii*, are highly modified, with an abbreviated stem and leaves reduced to scales. *In situ*, the adult plant shoots are usually covered with roots and organic detritus, which makes it difficult to observe the reduced stem or conduct any histology studies without damaging the plant. As a side note, a mistaken identification of a specimen of the leafless *Dendrophylax funalis* as *Cactus parasiticus* (Cactaceae) by Linnaeus (Fay and Chase, 2009) clearly illustrated the high degree of modification in the adult stages in these leafless orchids. Investigation of early seedling developmental stages in ghost orchids, therefore, could provide critical information on leafless orchid development.

Based on morphological features, we were able to characterize ghost orchid seed germination and seedling development into seven stages (Table 1). At stage 0, ungerminated seeds dimensions averaged 327 ×46 µm, which are smaller than those of its closest relative, *Dendrophylax varius* (∼500 µm), but seed morphology was very similar in the two species (Barthlott *et al.*, 2014). As the ghost orchid is an angraecoid (Vandeae, Orchidaceae) (Carlsward *et al.*, 2003, 2006*a*, *b*), its ungerminated seeds share similar morphological features to other Vanda seed types, including an elongated testa marginal ridge (Dressler, 1993). Ghost orchid protocorm development from stage 2 to stage 4 is similar to that described for other Vandeae members, including *Vanda* (Kauth *et al.*, 2008) and *Phalaenopsis* (Nishimura, 1981). We consider a ghost orchid protocorm, consisting of a swollen embryo hypocotyl structure bearing a dorsal crest, shoot meristem and first root meristem, to represent the mature embryo stage. Protocorms germinated from mature seeds and attaining stage 3 and later have been considered seedlings (Batygina *et al.*, 2003; Rasmussen *et al.*, 2015). Seedling development is associated with formation and elongation of a dorsal crest structure (Veyret, 1974; Pridgeon *et al.*, 1999), also considered a first leaf by Nishimura (1981). Stage 5 was defined by the emergence of root tips or shoots from one side of the protocorm. Shoots could become partially exposed, as seen by the presence of protruding leaves. However, protocorms with protruding shoots were not frequently seen at this stage. Shoot meristems with leaves, more often, were enclosed within the protocorm structure. In stage 6, roots were elongated with development of a distinct velamen layer.

In stage 6, a well-defined vascular connection between the protocorm dorsal crest and shoot was noted. This connection suggested that ghost orchid protocorms serve initially as photosynthetic organs during early development, similar to other orchids (Vinogradova and Andronova, 2002). *In situ*, ghost orchid protocorms are larger than those produced *in vitro*. They remain green and do not degenerate until the second root develops and becomes attached to the host substrate (E. Mujica, Orquideario Soroa, Pinar del Rio, Cuba, pers. comm.). Protocorm photosynthetic capacity has been documented in different epiphytic orchids, such as *Dendrobium*, *Vanda* and *Spathoglottis* (Hew and Khoo, 1980). Ghost orchid protocorm persistence *in situ*, therefore, could be critical until the photosynthetic root system is fully established (Veyret, 1965; Vinogradova and Andronova, 2002).

**Fig. 10:**
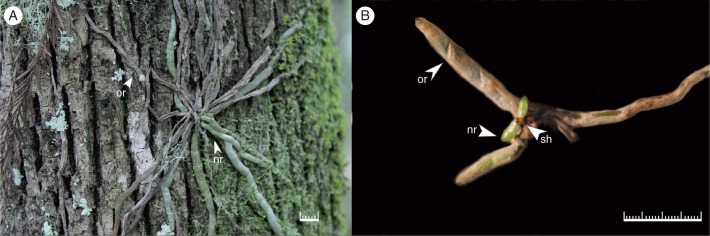
Ghost orchid growth habit *in situ* and in the greenhouse. (A) Ghost orchid *in situ* at FPNWR with old roots (or) above new roots (nr). (B) Greenhouse-acclimatized seedling with new roots derived from the apical shoot (sh) above substrate surface. Scale bar = 1 cm.

Anatomical examination of early seedling development provided an excellent opportunity to further investigate the abbreviated stem structure in the ghost orchid. Ghost orchid leaves remain reduced to scales even in mature seedlings (Figs 3, 4 and 6B). Among the Florida leafless orchids, the ghost orchid has leaf reduction similar to that of *Dendrophylax porrectus* seedlings, but different from that of *Campylocentrum pachyrrhizum* in that caducous leaves are produced in culture (N.H. Hoang and M. E. Kane, University of Florida, Gainesville, USA, unpubl. res.). Advanced seedling development in the ghost orchid is indicated by root formation subtending the shoot apical meristem, with these new roots emerging adjacent to the apical shoot, forming a monopodial stem (Fig. 6A, B). Development of new roots below the shoot apices in Vandeae species has been documented by others (Goh, 1983; Stewart *et al.*, 2006; Alrich and Higgins, 2008). Interestingly, new ghost orchid roots formed *in situ* emerge from beneath older roots at the apices of the reduced shoots (Fig. 10A), indicating that ghost orchid apical shoots in the wild are located underneath the root mass and are appressed to the phorophyte bark surface. This unique growth pattern has not been documented in the Orchidaceae before.

Following reintroduction, we have observed that *ex vitro* seedlings planted with the shoot apex facing outward produce new unattached aerial roots above old roots (Fig. 10B), instead of attaching to the host tree surface, where the microhabitat is more supportive for root growth. Ghost orchid roots attached to their host plants exhibit dorsiventral symmetry (Chomicki *et al.*, 2014), while aerial unattached roots display radial symmetry. The degree to which the velamen layer in these unattached roots can absorb water and nutrients, or develop any mycorrhizal associations, is not clear. The capacity for aerial roots to absorb water and mineral nutrients has been reported (Zotz and Winkler, 2013). However, aerial roots in *Epidendrum*, *Phalaenopsis*, *Vanda* and a few other species have a velamen layer that is impermeable to water and mineral nutrients (Dycus and Knudson, 1957). Whether the production of unattached aerial roots, caused by outplanting direction (outward or towards the host substrate surface), could affect the survival rate of *ex vitro* plants under greenhouse conditions or in the field deserves further investigation.

The capacity of mature ghost orchid seedlings to develop elongated inflorescences *in vitro* (Fig. 1B), morphologically similar to *in situ* inflorescences (Fig. 1C), is remarkable. Although inflorescence production *in vitro* was limited to a very few seedlings, it provided an opportunity to investigate ghost orchid inflorescence development. After 2–3 months most inflorescences produced *in vitro* browned and died. This response could be similar to the shoot-tip necrosis observed in many species that was due to calcium or boron deficiency (reviewed by Bairu *et al.*, 2009). Inflorescence development was also influenced by genotype, as several seedlings produced inflorescences that remained vegetative and displayed indeterminate growth (Fig. 6C). The absence of flower buds *in vitro* suggests that the inductive factors promoting caulescent shoot (inflorescence) formation and actual floral bud development may differ (Teixeira da Silva *et al.*, 2013). Flowering of plants in the wild is sporadic, and nothing is known regarding the environmental and physiological factors required for floral induction and development. Screening for both environmental conditions and culture medium components that promote flowering *in vitro* on mature seedlings could prove useful in elucidating the factors controlling ghost orchid flowering.

Although it is considered very common in *Dendrophylax funalis* and *Dendrophylax* *fawcettii* (Whitten and Carlsward, 2006), keiki production has not been observed *in situ* on *D. lindenii*. Inflorescences originate from the underside of the plant *in situ* and produce three or four nodes, each covered with non-photosynthetic bracts, before terminating in a flower (Fig. 1B, C). Inflorescence branching from the upper nodes has been observed *in situ* and now under *in vitro* growing conditions. Given that keikis (plantlets) can be generated from excised inflorescence buds cultured under *in vitro* conditions (data not shown), these vegetative buds (Fig. 6D) could serve as an explant source for ghost orchid clonal propagation, similar to methods developed for *Phalaenopsis* (Tokuhara and Mii, 1993; Tanaka *et al.*, 1988).

### Comparative symbiotic seed germination

Given that the two *Ceratobasidium* mycobiont strains were isolated from mature plants *in situ*, it could be argued that *D.* *lindenii* plants are partially mycoheterotrophic at maturity. However, it remains unclear whether this orchid utilizes *Ceratobasidium* or other *Rhizoctonia*-like fungi to facilitate seed germination and seedling development *in situ*. While efforts are currently under way to recover other mycorrhizal fungi from *D. lindenii* seedlings in South Florida, we attempted to answer this question, in part, by conducting *in vitro* symbiotic seed germination experiments using two *Ceratobasidium* strains (Dlin-379 and Dlin-394). The isolation of orchid germination mycobionts from mature orchid roots has been reported (Rasmussen, 1995; Smith and Read, 1996; Otero *et al.*, 2004; Porras-Alfaro and Bayman, 2007; Rasmussen *et al.*, 2015). However, these two ghost orchid mycobiont strains had significantly different effects on seed germination. *Dendrophylax lindenii* seeds failed to germinate on oatmeal agar medium alone or during symbiotic seed culture with strain Dlin-379. Symbiotic culture with *Ceratobasidium* Dlin-394 promoted *in vitro* seed germination (Fig. 8) and seedling maturation. However, whether the Dlin-394 *Ceratobasidium* strain serves as a germination mycobiont *in situ* and whether this association persists through plant maturation requires further study. In orchids, fungal pelotons are digested and serve as a nutrient source (Rasmussen, 1995; Rasmussen *et al.*, 2015). Chomicki *et al.* (2014) have demonstrated that fungal pelotons in mature ghost orchid roots are digested. The presence of permanent mycobionts during orchid reintroduction is critical (Zettler *et al.*, 2003). In the case of *D. lindenii*, the availability of *Ceratobasidium* Dlin-394 at reintroduction sites could be important both to sustain newly reintroduced plants and to promote seed germination in the following generations.

Ceratobasidiaceae members are known to promote seed germination in a wide range of orchid subfamilies, specifically Apostasiodeae, Vanilloideae, Epidendroideae, Orchidoideae (Dressler, 1993; Rasmussen *et al.*, 2015) and Vandeae (Irawati, 2009; Rasmussen and Rasmussen, 2014). Currently, there is only one published study on *Thanatephorus* (Ceratobasidiaceae) germination promotion capacity in seeds of *Taeniophyllum obtusum*, also a leafless orchid species (Irawati, 2009). Our result confirmed *Ceratobasidium* as a germination mycobiont of the Angracinae subtribe (Vandeae).

Why the other *Ceratobasidium* strain (Dlin-379), isolated a year earlier, had little effect on ghost orchid germination remains unresolved. However, culture conditions may influence the mycorrhizal capacity of fungal isolates. In a preliminary symbiotic seed culture experiment conducted in the dark, the Dlin-379 strain exhibited the capacity to infect seed and form pelotons in ghost orchid protocorms (N.H. Hoang and M. E. Kane, University of Florida, Gainesville, USA, unpubl. res.). While experimental conditions were different, loss of fungal activity should be taken into account. *Rhizoctonia* spp. apparently lose their ability to establish orchid mycorrhizae in pure culture, as Rasmussen (1995) reported. Similarly, Alexander and Hadley (1984) noted a decline in mycorrhizal capacity within just 2 years for cultures stored on PDA at 4 °C. Many significant strains of *Rhizoctonia*-like fungi have been safeguarded worldwide by means of cryopreservation (e.g. UAMH), but studies are needed to determine whether fungi preserved in this manner also experience a decline in their ability to germinate orchid seeds. Moreover, some strains of *Ceratobasidium* have proved difficult to revive from cryopreservation compared with other types of *Rhizoctonia*-like fungi (L. Sigler, University of Alberta, Alberta, Canada, pers. comm.).

We also noted that the germination percentage of seed on OM/Dlin-394 decreased at week 10. After week 10, all ghost orchid seedlings were transferred to fresh medium. Seedlings from P723 media continued to develop normally, while seedlings from OM medium infected with Dlin-394 exhibited significant variability in growth between seedlings (Fig. 7D). This variability could be due to genotypic differences between seedlings or to the rapid growth of Dlin-394 under *in vitro* culture conditions, as reflected in Fig. 2. Although ghost orchid seed availability *in situ* is very low, additional symbiotic culture studies using seed collected from multiple populations could provide insight into the influence of genotype and possibly ecotypic differentiation.

Protocorms produced using different germination methods display morphological variation. The protocorms produced on OM media with Dlin-394 appeared slightly bigger, light green and hyperhydric, which differed from protocorms cultured on P723 medium (Fig. 7B). Sizes of *in vitro* protocorms (3–4 mm) are much smaller than those of *in situ* protocorms (8–10 mm), suggesting that the *in vitro* symbiotic germination conditions in this study, even with the supplement of Dlin-394, may not be optimal. Unsuitable media could be the same reason why germination percentages on asymbiotic media P723 were lower than on OM media supplemented with Dlin-394. Further research is needed to optimize asymbiotic germination media as well as understanding of the role of Dlin-394 in subsequent seedling development stages.

We have demonstrated that ghost orchid seeds can be asymbiotically germinated *in vitro* and that seedling development differs from that of leafy orchids mostly in the later seedling stages (stage 5 and 6) with highly reduced shoots, vestigial leaves and possibly different stem growth orientation. Symbiotic co-culture with its *Ceratobasidium* germination mycobiont (Dlin-394) enhances both seed germination rate and seedling development. Most importantly, our results provide opportunities to further examine the effects of mycobionts on subsequent *ex vitro* acclimatization, establishment and sustainability of reintroduced plants. With the roots being the sole photosynthetic organ, novel approaches are required to effectively attach this leafless orchid to its host trees without excluding light. These questions are currently being explored. Until this information is available, conservation of the ghost orchid natural areas within the Big Cypress Basin eco-region will remain crucial for generating spontaneous seedlings of *D.* *lindenii* and other orchids faced with extinction, at least in the foreseeable future.
